# Functional cooperation between ATG7/autophagy and the PALB2 tumor suppressor in mitochondrial regulation, redox homeostasis, and neuronal health

**DOI:** 10.1080/27694127.2022.2080314

**Published:** 2022-05-29

**Authors:** Yanying Huo, Akshada Sawant, Eileen White, Bing Xia

**Affiliations:** Rutgers Cancer Institute of New Jersey, 195 Little Albany Street, New Brunswick, NJ 08903, USA

## Abstract

In a recently published study, we described a genetic interaction and functional cooperation between *Atg7*, an essential autophagy gene, and *Palb2*, a breast cancer susceptibility gene, in the maintenance of mitochondrial function, redox homeostasis, and neuronal viability. Our studies uncovered a new function for PALB2 in mitochondrial regulation and provided new insights into the role of ATG7/autophagy in maintaining redox balance and neuronal health. Here, we summarize the main findings and discuss their implications in neurodegeneration and the potential treatment of *PALB2* mutant and similar cancers with autophagy inhibition.

Autophagy plays key roles in the pathogenesis of cancer and neurodegenerative diseases. In cancer, autophagy promotes metabolic fitness through recycling and suppresses anti-tumor immune responses by suppressing inflammation. In the central nervous system, autophagy facilitates the proper function and survival of neurons, largely by clearing toxic wastes inside the cells and preventing excessive production of reactive oxygen species (ROS). As neurons are post-mitotic (non-proliferating) and thereby unable to dilute wastes through cell division, and highly sensitive to oxidative stress, loss of autophagy leads to neuronal death and neurodegeneration.

Breast cancer is a common disease with about 10% arising from families with inherited mutations in susceptibility genes such as *BRCA1, BRCA2* and *PALB2*, etc. These genes encode proteins that play key roles in the repair and cellular response to DNA damage, especially DNA double-strand breaks (DSBs). Additionally, both BRCA1 and PALB2 promote cellular redox homeostasis. In 2013, we reported that monoallelic loss of the essential autophagy gene *Becn1* delays spontaneous mammary tumor formation following conditional knockout (CKO) of *Palb2* in mice. More recently, in 2018 Jun-Lin Guan’s lab reported that combined CKO of another essential autophagy gene, *Rb1cc1/Fip200*, with *Brca1* (and *Trp53*) also delays mammary tumorigenesis. Given that loss of BRCA1 and PALB2 leads to increased DNA damage and oxidative stress, the above results strongly suggest that autophagy facilitates the development of cancers with these characteristics.

In a recent paper published in PLOS Genetics, we reported an unexpected role for PALB2 and the genetic interactions between *Palb2* and another essential autophagy gene, *Atg7*, in the brain [1]. To better understand of the role of autophagy in familial breast cancer development, we generated mouse models with individual and combined CKO of *Palb2* and *Atg7* using *Wap-cre*, which is mainly expressed in secretory mammary epithelial cells and is a commonly used deleter for breast cancer mouse models. Although leaky expression of *Wap-cre* has been reported in the brain, to our knowledge it has not been shown to hamper breast cancer studies. Unexpectedly, *Wap-cre* causes highly efficient deletions of the genes in the brain, and mice with *Atg7* deletion manifest symptoms of neurodegeneration, such as tremor and impaired balance, from approximately 3 months of age. Interestingly, combined deletion of *Palb2* and *Atg7* leads to accelerated and exacerbated neurodegenerative phenotypes compared with *Atg7* deletion alone. In fact, the double CKO mice practically all succumb to neurodegeneration prior to any mammary tumor development, preventing any assessment of *Atg7* in mammary tumorigenesis induced by inactivation of *Palb2*.

Analyses of brain histopathology confirmed the requirement of ATG7/autophagy for the viability of Purkinje neurons in the cerebellum that control balance and certain other aspects of motor function, as well as a later loss of dopaminergic neurons in the substantia nigra, which play key roles in motor planning and movement, in mice deleted of *Atg7*. Notably, combined loss of ATG7 and PALB2 leads to even more rapid loss of Purkinje cells; moreover, significant apoptosis in the cerebellar granule cell layer is detected in the double CKO mice at younger ages. These findings largely explain the accelerated and severe neurodegenerative phenotypes.

Immunohistochemistry (IHC) analyses revealed that loss of PALB2 leads to increased γH2AX, a marker of DSBs, in Purkinje cells, while any increase in γH2AX in *atg7*-deleted cells is modest. Interestingly, *palb2*-deleted Purkinje cells also show much stronger 8-oxo-dG, suggestive of high levels of DNA oxidation, whereas in *atg7*-deleted mice the entire cerebellum, especially the molecular layer, exhibits much stronger 4-HNE signals, indicative of lipid peroxidation. These findings suggest that PALB2 and ATG7 regulate different aspects of DNA repair and redox homeostasis so that combined loss of the two would cause stronger adverse effects that lead to a rapid loss of Purkinje cells and some other cell types in the granule cell layer of the cerebellum, leading to severe motor deficits.

In addition to the above tissue-specific CKO mice, we also created adult whole-body knockout (WBKO) mice using the tamoxifen-inducible *Ubc-CreERT2* as a driver. As reported previously, such conditional, systemic loss of ATG7 in adult mice is lethal within 2-3 months predominantly due to neurodegeneration. Again, mice with combined WBKO of *Palb2* and *Atg7* show accelerated onset and increased severity of the neurodegenerative phenotypes, which further shortens survival. Interestingly, feeding the double WBKO mice with water containing N-acetylcysteine/NAC, an ROS scavenger, significantly rescues Purkinje cell loss and prolonged survival, suggesting that excessive ROS is likely the major cause of severe neurodegeneration in these mice. This scenario was further supported by the finding that unlike *Palb2*, combined deletion of *Brca2* with *Atg7* does not accelerate neurodegeneration, even though BRCA2 and PALB2 function together in DNA repair. Note that unlike PALB2, BRCA2 does not appear to play a role in redox regulation.

Serendipitously, this study uncovered a novel role for PALB2 in the regulation of mitochondrial gene expression and biogenesis. Motivated by the well-established role of autophagy in mitochondrial quality control, we analyzed several mitochondrial proteins in the brain sections, and we found that loss of PALB2 causes marked increase in the levels of these proteins, again especially evident in Purkinje cells. This was further confirmed in human DAOY medulloblastoma cells, which are of a cerebellum origin, upon *PALB2* knockdown and knockout. Comparing the effects of *PALB2* and *ATG7* KO in the DAOY cells, loss of PALB2 causes increased cellular ROS as detected by DCF-DA, which is mainly oxidized by hydrogen peroxide, whereas *ATG7* KO leads to increased mitochondrial superoxide as assayed by MitoSOX Red. Moreover, KO of either *PALB2* or *ATG7* leads to decreased mitochondrial oxidative phosphorylation, and combined knockout of the two results in an even stronger reduction. Together, these data again demonstrate that PALB2 and ATG7 regulate different aspects of mitochondrial biogenesis, quality, and function, therefore simultaneous loss of the two causes stronger effects.

Overall, our studies revealed a new function for PALB2 and provide further insight into the role of ATG7 in mitochondrial quality control, suppression of lipid peroxidation, and sustenance of neuronal viability. Although our planned assessment of mammary cancer was not possible due to early-onset neurodegeneration, the results still predict that *Atg7* ablation may impair the fitness and survival of *Palb2* mutant cancer cells, thereby delaying or preventing tumor development. Such an outcome would lend further support to the potential use of autophagy inhibition to treat *PALB2* and *BRCA1* mutant cancers and possibly other cancers associated with DNA damage and oxidative stress. To this end, it should be noted that in the case of *PALB2* or *BRCA1* mutant cancers, the patients are mostly heterozygous mutation carriers and only tumor cells have further lost or mutated the WT allele and become functionally null; therefore, autophagy inhibition can be expected to be more toxic to tumor cells than normal cells, including neurons. Moreover, potential neurological side effects may be avoided by using therapies that do not cross the blood-brain barrier.
